# Time trends and birth rates in women with congenital heart disease; a nationwide cohort study from Norway 1994–2014

**DOI:** 10.1016/j.ijcchd.2024.100507

**Published:** 2024-03-30

**Authors:** Marit Sandberg, Tatiana Fomina, Ferenc Macsali, Gottfried Greve, Mette-Elise Estensen, Nina Øyen, Elisabeth Leirgul

**Affiliations:** a Department of Clinical Science, University of Bergen, Norway; b Department of Obstetrics and Gynecology, Haukeland University Hospital, Bergen, Norway; c Department of Global Public Health and Primary Care, University of Bergen, Norway; d Norwegian Institute of Public Health, Norway; e Department of Heart Disease, Haukeland University Hospital, Bergen, Norway; f Department of Cardiology, Oslo University Hospital, Oslo, Norway; g Department of Medical Genetics, Haukeland University Hospital, Bergen, Norway

**Keywords:** Congenital heart disease, Prevalence, Birth rate, Fertility, Reproductive health, Epidemiology

## Abstract

**Background:**

More women with congenital heart disease (CHD) reach reproductive age, but little is known of their success in having children. We investigated time trends of CHD in women of reproductive age and maternal CHD in childbirth and compared birth rates in women with CHD to birth rates in women without heart disease.

**Methods and results:**

In a national cohort, we combined information from five registries in Norway 1994–2014. Among 1,644,650 women aged 15–45 years, 5672 had CHD. Among 1,183,851 childbirths, 3504 were registered with maternal CHD. The prevalences of mild and moderate/severe CHD in women increased by an average of 3–4% per year 1994–2014, as did the prevalences of mild and moderate/severe maternal CHD in childbirth. Compared to women without heart disease, the likelihood of having children was similar for women with mild CHD (rate ratio 1.03, 95% confidence interval 0.97–1.09) but lower for women with moderate/severe CHD (rate ratio 0.75, 95% confidence interval 0.68–0.84). The mean number of childbirths was similar in women with mild CHD and women without heart disease (1.81 vs 1.80, p = 0.722) but lower in women with moderate/severe CHD (1.42, p < 0.001).

**Conclusion:**

In a national cohort over two decades of women of reproductive age, the prevalence of maternal CHD in childbirth reflected the increasing prevalence of CHD in the population. Birth rates were similar for women with mild CHD and women without heart disease, whereas women with moderate/severe CHD were less likely to have children and had a lower mean number of childbirths.

## Highlights

• More women with congenital heart disease (CHD) reach reproductive age.
• The prevalence of CHD in women (15–45 years) increased 3–4% annually from 1994 to 2014.
• The prevalence of maternal CHD in childbirth increased 3–4% annually from 1994 to 2014.
• Women with mild CHD had similar birth rates to women without CHD.
• Women with moderate/severe CHD had lower birth rates than women without CHD.

## Graphical abstract

**Figure:**
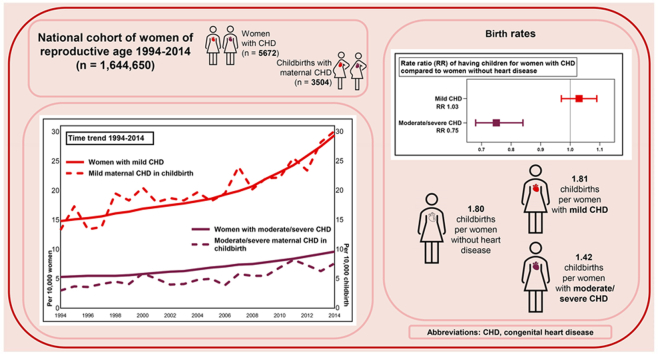

## 1 Introduction

Congenital heart disease (CHD) is the most frequent birth defect, reported to affect 5 to 12 per 1000 live births [1,2]. The birth prevalence of CHD has increased worldwide since the 1970s [2], most likely explained by advancements in diagnostic tools for detecting mild heart defects and better registration of such defects [3]. In parallel, significant advantages in postnatal care, cardiac surgery, and medical follow-up have improved survival for individuals with severe heart defects [4], contributing to the increasing prevalence of CHD in adults.

CHD represents a broad spectrum of severities, from conditions with minor clinical impact on daily life to life-threatening conditions requiring extensive and repeated surgery in childhood and adolescence. Anyhow, CHD is a lifelong chronic condition with implications for health and quality of life [5,6].

Pregnancy leads to major hemodynamic changes even in normal pregnancies [7]; therefore, some women with CHD have been advised against childbearing due to concerns about maternal cardiac health. During the late 1990s and 2000s, international efforts led to the development of risk scores and guidelines for pregnancies in women with CHD, and there is now increasing evidence that many women with CHD can complete the pregnancy with low maternal risk [[8], [9], [10]]. Still, maternal CHD is associated with increased risks of adverse maternal, obstetric, and neonatal outcomes compared to the general population [11]. To optimize the outcomes for mother and child, pre-pregnancy counseling and multidisciplinary management of pregnancy and delivery are recommended, especially in pregnancies with moderate to severe maternal CHD [11]. For healthcare planning, knowledge of the prevalence of maternal CHD in childbirth is therefore highly relevant for healthcare authorities.

On an individual level, information on the prospects of having children is essential for young women with CHD. Guidelines recommend addressing fertility in pre-pregnancy counseling for women with CHD [11]. However, besides reports on impaired fertility in women with Fontan circulation [11], knowledge of fertility in women with CHD is sparse. Beyond the biological ability to conceive and complete a pregnancy, a woman's prospects of having children depend on her physical health, psychosocial conditions, and her attitude towards childbearing. These factors can all be influenced by growing up and living with a CHD [5].

In a national cohort of women of reproductive age (15–45 years), we addressed the following objectives: First, we investigated the prevalence of CHD from 1994 to 2014 and reflected this to the prevalence of maternal CHD in childbirth in the same period. Second, we examined birth rates (the likelihood of having children and the mean number of childbirths) in women with CHD compared to women without heart disease.

## 2 Materials and methods

### 2.1 Data sources

The unique personal identification number assigned to all residents of Norway enables data linkage between national registries and data sources. The Norwegian National Registry and Statistics Norway contains demographic data on all residents. The Medical Birth Registry of Norway (MBRN) has since 1967 registered information on all births in Norway, including maternal health before and during pregnancy [12]. The Research in Hospital Register collected data on all discharge diagnoses and procedures for individuals with cardiovascular disease diagnoses from somatic hospitals in Norway 1994–2009 [13]. The Norwegian Patient Registry includes information on diagnoses and procedures from inpatient and outpatient treatment in the specialist health service since 2008. The Cause of Death Registry records the causes of death from death certificates.

### 2.2 Study population

First, we identified all women aged 15–45 in Norway between January 01, 1994, and December 31, 2014. Second, we found all childbirths of these women registered in MBRN between January 01, 1994, and December 31, 2014. We excluded women with a diagnosis of patent arterial duct only, women with an unspecified CHD recorded in the MBRN form at birth but no further registration of CHD, women with syndromes more often associated with CHD and with possibly reduced reproductive potential (Down, Turner, Noonan and DiGeorge syndrome), and women with acquired heart disease before the age of 45 years (ischemic heart disease, heart failure, cardiomyopathy, arrhythmia, valve disease or pulmonary hypertension) and no diagnosis of CHD.

### 2.3 Case ascertainment and classification of CHD

As CHD is a lifelong condition, women were classified with CHD regardless of whether the diagnosis was registered before or after their pregnancy. Information on CHD was retrieved from the registries mentioned in section 2.1 by diagnosis codes for cardiac malformations in the International Classification of Diseases, Eighth, Ninth, and Tenth Revisions and by cardiac procedure codes by the NOMESCO Classification of Surgical Procedures and the Norwegian Classification of medical procedures; 3rd edition, as described in a previous article [14].

For females before the age of 18 years, we used all the recorded CHD diagnoses. For women 18 years or older, we only registered their CHD diagnoses as such if their first-time CHD diagnoses were assigned in the following departments: pediatric, cardiology, thoracic surgery, or internal medicine. For women 30 years or older, we did not count the diagnosis of congenital valve defect if an acquired valve defect had been diagnosed before.

If a woman had been recorded with successive diagnoses, we prioritized the source of diagnosis as follows: first entry at a university hospital, first entry at a regional/local hospital, MBRN, the Cause of Death Registry, procedure codes from university hospitals, and procedures codes from regional/local hospitals.

We assigned every woman to a single cardiac phenotype. The recorded diagnoses and procedures from different classification systems were translated into cardiac phenotypes in a hierarchical approach using an algorithm developed previously for analyses of Norwegian and Danish register data [14]. Combined defects were coded according to the embryological first appearing condition, usually representing the more complex defect.

Women with CHD were subsequently divided into subgroups of mild, moderate, severe, and other CHD to resemble the classification of congenital heart disease by the European Society of Cardiology (Table 1) [6]. Due to a lack of clinical information and surgical history, we merged moderate and severe CHD into one group.

**Table 1:** Classification of mild CHD, moderate/severe CHD, and other CHD^a^.

Mild CHD	Atrial septal defect, ventricular septal defect, or selected valve defects (mitral or aortic insufficiency, pulmonary stenosis or insufficiency, or unspecified anomaly of the heart valves) not combined with defects of higher priority in our classification system.
Moderate/severe CHD	Heterotaxia with other heart defects, truncus arteriosus, transposition of the great arteries, tetralogy of Fallot, double outlet right ventricle, interrupted aortic arch, atrioventricular septal defect, total or partial anomalous pulmonary venous return, hypoplastic left heart syndrome, mitral stenosis/atresia, coarctation of aorta, supra valvular aorta stenosis, valvular aortic stenosis, hypoplastic right heart syndrome, tricuspid atresia, pulmonary atresia, valvular pulmonary atresia, Ebstein anomaly, or congenitally corrected transposition of the great arteries. In addition, women with procedures indicating Fontan circulation or mechanical valve replacement or diagnosis indicating CHD with pulmonary hypertension were included in this group.
Other CHD	Coronary malformation or not otherwise specified malformations of the heart, the great veins, or the great arteries not combined with defects of higher priority in our classification system.

Abbreviations: CHD, congenital heart disease.

a Further information on CHD is provided in Section 2.3, Case ascertainment and classifications of CHD.

### 2.4 Characteristics

We retrieved information on country of origin, year of birth, marital status by the end of 2014, and years of education by the end of 2014 from Statistics Norway.

### 2.5 Pregnancy outcome

Childbirths were defined as live births and stillbirths after 22nd gestational week.

### 2.6 Statistical analyses

The prevalence of CHD was reported per 10,000 women of age 15–45 years per calendar year. The prevalence of maternal CHD in childbirth was reported per 10,000 births per calendar year. We estimated the average annual percentage change (AAPC) [15] in prevalence with a 95% confidence interval (CI) using Joinpoint Regression Program [16].

We calculated rates of having children as numbers of women with at least one childbirth during 1994–2014 divided by the total person-years of women. All women were followed from age 15 years until first childbirth, age 45 years, death, or end of the study period December 31, 2014 (whichever came first). The association between maternal CHD and having children was estimated by the rate of having children among women with CHD compared to women without heart disease using Poisson regression analyses to calculate a rate ratio with a 95% CI adjusted for the mother's birth year. We evaluated comorbidity as a confounder; however, comorbidity is likely an intermediate variable between the association of CHD in women and childbirth; therefore, we did not include comorbidity in the model.

Finally, we calculated the proportion of women of different CHD subgroups who had become mothers by age ≥40 years by December 31, 2014, and the mean number of childbirths per woman of this age. In this analysis, a woman's childbirths before 1994 were also counted. By student t-test, we compared the mean number of childbirths in women of different subgroups of CHD to the reference population.

To avoid detection bias in analyses comparing birth rates, we did not count maternal CHD registered in MBRN. A two-sided p-value of <0.05 was considered significant throughout the study.

The data linkage was performed with SAS version 9.4. Analyses were performed with STATA version 17 and the National Cancer Institutes’ Joinpoint Regression Program version 4.1.9.0. The study was approved by the Regional Ethics Committee of Western Norway (REK 111545).

## 3 Results

Among 1,668,032 women aged 15–45 years resident in Norway between 1994 and 2014, we sequentially excluded women with persistent ductus arteriosus (n = 374), unspecified CHD diagnosis from MBRN in the first year of life (n = 399), Down, Turner, DiGeorge, or Noonan syndrome (n = 859), and acquired heart disease (n = 21,750), leading to a study population of 1,644,650 women (Supplementary Fig. 1).

### 3.1 Characteristics

Compared to women without heart disease, women with CHD were younger, less often married/cohabitant, and had shorter education (Table 2).

**Table 2:** Characteristics in women with congenital heart disease and women without heart disease among 1,644,650 women of reproductive age (15–45 years) in Norway 1994–2014.

		Women without heart disease n = 1,638,978	Women with any CHD n = 5672	Women with mild CHD^a^ n = 3786	Women with moderate/severe CHD^a^ n = 1281	Women with other CHD^a^ n = 605
**Country of origin** **n (%)**	Norway	1,383,229 (84.4)	5115 (90.2)	3421 (90.4)	1152 (89.9)	542 (89.6)
	Other	255,749 (15.6)	557 (9.8)	365 (9.6)	129 (10.1)	63 (10.4)
**Year of birth** **n (%)**	1949–1965	537,697 (32.8)	1020 (18.0)	673 (17.8)	245 (19.8)	93 (15.4)
	1966–1982	570,461 (34.8)	1612 (28.4)	1030 (27.2)	359 (20.0)	223 (36.9)
	1983–1999	530,820 (32.4)	3040 (53.6)	2083 (55.0)	668 (52.2)	289 (47.8)
**Marital status**^b^ **n (%)**	Married/cohabitant	917,650 (56.0)	2371 (41.8)	1583 (41.8)	503 (39.3)	285 (47.1)
	Other	721,328 (44.0)	3301 (58.2)	2203 (58.2)	778 (60.7)	320 (52.9)
**Education**^b^ **n (%)**	<10 years	389,471 (23.8)	1961 (34.6)	1314 (34.7)	439 (34.3)	208 (34.4)
	11–13 years	563,931 (34.4)	1652 (29.1)	1094 (28.9)	394 (30.8)	164 (27.1)
	>14 years	603,367 (36.8)	1556 (27.4)	1060 (28.0)	236 (25.5)	170 (28.1)
	Missing	82,209 (5.0)	503 (8.9)	318 (8.4)	122 (9.5)	63 (10.4)

Abbreviations: CHD, congenital heart disease.

a Mild CHD: Septal defects, mitral or aortic insufficiency, pulmonary stenosis or insufficiency, or unspecified anomaly of the heart valves. Moderate/severe CHD: Heterotaxia with other heart defects, truncus arteriosus, transposition of the great arteries, tetralogy of Fallot, double outlet right ventricle, supra valvular aorta stenosis, interrupted aortic arch, atrioventricular septal defect, total or partial anomalous pulmonary venous return, hypoplastic left heart syndrome, mitral stenosis/atresia, coarctation of aorta, valvular aortic stenosis, hypoplastic right heart syndrome, tricuspid atresia, pulmonary atresia, valvular pulmonary atresia, Ebstein anomaly, congenitally corrected transposition of the great arteries, Fontan circulation, mechanical valves or heart defects with pulmonary hypertension. Other CHD: Coronary malformation and not otherwise specified malformations of the heart, the great veins, or the great arteries.

b Status by 2014.

### 3.2 Prevalence of CHD in women aged 15–45 years

Among 1,644,650 women aged 15–45 years, 5672 women were classified with CHD (34.5 per 10,000). 3786 women were classified with mild CHD, 1281 with moderate/severe CHD, and 605 with other CHD. Within the group of moderate/severe CHD, one out of four was likely to be severe CHD, according to the European Society of Cardiology classification [6]. From 1994 to 2014 the prevalence of mild CHD in women aged 15–45 years rose from 14.8 to 29.4 per 10,000 (AAPC 3.4%, 95% CI 3.0–3.8) and the prevalence of moderate/severe CHD rose from 5.3 to 9.6 per 10,000 (AAPC 3.1%, 95% CI 2.9–3.4) (Fig. 1).

**Fig. 1:**
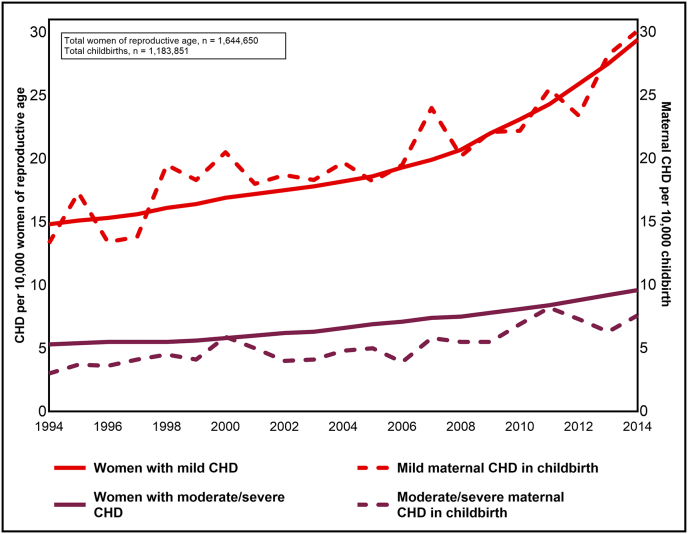
Prevalence of mild CHD (upper line) and prevalence of moderate/severe CHD (lower line) per 10,000 women of reproductive age (15–45 years) (left y-axis), and prevalence of mild maternal CHD (upper dashed line) and prevalence of moderate/severe CHD (lower dashed line) per 10,000 childbirths (right y-axis), in Norway, 1994–2014. Abbreviations: CHD, congenital heart disease.

### 3.3 Prevalence of maternal CHD in childbirths

The women in the study population had 1,183,851 childbirths between 1994 and 2014. Among the 3504 childbirths identified with maternal CHD (29.6 per 10,000), 2382 were with mild CHD, 612 with moderate/severe CHD, and 510 with other CHD. From 1994 to 2014, the prevalence of mild maternal CHD in childbirth rose from 13.3 to 20.2 per 10,000 (AAPC 3.1%, 95% CI 2.3–3.9), and the prevalence of moderate/severe maternal CHD rose from 3.0 to 5.2 per 10,000 (AAPC 3.9%, 95% CI 2.8–5.1).

### 3.4 Rate ratio of having children

**Fig. 2:**
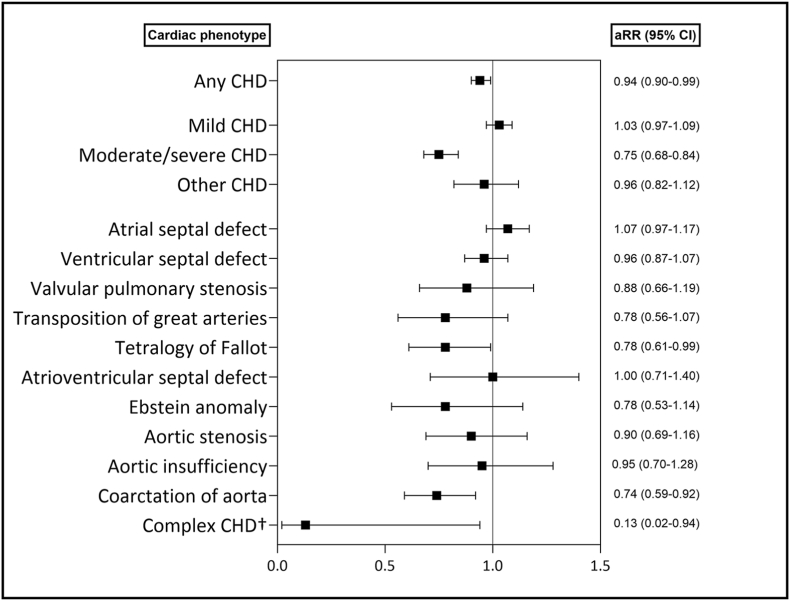
Rate ratio of having children in women of reproductive age (15–45 years) with phenotypes of congenital heart disease compared to women without heart disease, adjusted for mother's birth year, in Norway 1994–2014. Abbreviations: CHD, congenital heart disease; aRR, adjusted rate ratio; CI, confidence interval. †Complex CHD consists of double outlet right ventricle, double outlet left ventricle, double inlet chamber, hypoplastic right heart syndrome, hypoplastic left heart syndrome, Fontan circulation, and mechanical valve replacements.

Compared to women without heart disease, the adjusted rate ratio (aRR) of having children for women with mild CHD was 1.03 (95% CI 0.97–1.09), and for women with moderate/severe CHD, 0.75 (95% CI 0.68–0.84). Investigating more specific cardiac phenotypes demonstrated major diversity where atrial septal defects had aRR 1.07 (95% CI 0.97–1.17) and ventricular septal defects aRR 0.96 (95% CI 0.87–1.07), whereas transposition of the great arteries had aRR 0.78 (95% CI.0.56–1.07), tetralogy of Fallot aRR 0.78 (95% CI.0.61–0.99), aortic stenosis aRR 0.90 (95% CI.0.69–1.16) and complex CHD had aRR 0.13 (95% CI.0.02–0.94) (Fig. 2).

### 3.5 Mean number of childbirths

The proportions of women who became mothers by the age of ≥40 years were 76.8% for women without heart disease, 77.1% for women with mild CHD, and 63.6% for those with moderate/severe CHD (Table 3). The mean numbers of childbirths were 1.80 (reference) for women without heart disease, 1.81 (p = 0.722) for women with mild CHD, and 1.42 (p < 0.001) for women with moderate/severe CHD. Among women with at least one childbirth, there was no difference between women with moderate/severe CHD and those without heart disease (2.24 vs. 2.35 childbirths per woman, p = 0.073).

**Table 3:** Mean number of childbirths in women and mothers^a^ of age ≥40 years by December 31, 2014, without heart disease and with congenital heart disease.

	Women who became mothers^a^ n (%)	Mean number of childbirths in women (95% CI)	P-value	Mean number of childbirths in mothers^a^ (95% CI)	P-value
Women without heart disease (n = 854,989)	656,165 (76.8)	1.80 (1.79–1.80)	(ref)	2.35 (2.34–2.34)	(ref)
Women with mild CHD (n = 1082)	834 (77.1)	1.81 (1.73–1.89)	0.722	2.35 (2.28–2.42)	0.814
Women with moderate/severe CHD (n = 423)	269 (63.6)	1.42 (1.30–1.55)	<0.001	2.24 (2.13–2.35)	0.073

Abbreviations: CHD, congenital heart disease.

a Mothers are women who have had at least one childbirth.

## 4 Discussion

In this nationwide cohort study, the prevalences of mild and moderate/severe CHD in women aged 15–45 years increased by an average of 3–4% per year during 1994–2014. Similarly, the prevalences of mild and moderate/severe maternal CHD in childbirth increased by 3–4% annually in the same period. Compared to women without heart disease, women with moderate/severe CHD were less likely to have children and had a lower mean number of childbirths. We found no differences in birth rates between women with mild CHD and women without heart disease.

The increased prevalence of CHD in women of reproductive age is in accordance with the previously reported rise in the prevalence of adults living with CHD [17,18]. The prevalence of maternal CHD in childbirth is less described. Still, a national U.S. study demonstrated that the prevalence of maternal CHD in childbirth increased from 4.2 to 10.9 per 10,000 childbirths between 2000 and 2018, resulting in an AAPC of 4.8% [19].

Our findings of a considerably higher prevalence of maternal CHD (29.6 per 10,000 childbirths) could reflect the somewhat lower birth prevalence of CHD in North America compared to Europe [1]. More importantly, our study had a virtually complete registration of CHD in women and maternal CHD in childbirth, while the U.S. study registered maternal CHD from discharge diagnoses of the delivery hospitalization only, and milder CHD with low clinical impact in pregnancy may have been missed.

Results from studies regarding birth rates in women with mild CHD are limited and conflicting. A cohort study of women aged 16–45 years from Taiwan demonstrated lower live birth rates in women with simple CHD compared to an age-matched control group [20], anda Danish cohort study reported that fewer women aged 34–41 years with mild CHD had children relative to women without CHD (74.0% vs. 80.4%) [21]. However, supporting our findings of similar birth rates, another Danish cohort study found no difference in rates of having children between women with mild CHD like septal defects compared to women without heart disease [22]. For women with more severe CHD, our results of lower birth rates in women with moderate/severe CHD compared to women without heart disease are consistent with conclusions from all the abovementioned studies [[20], [21], [22]].

In women with complex and cyanotic CHD, there are reports of irregular menstruation [23,24] and lower anti-Müllerian hormone, a biomarker that indicates ovarian function [25], suggesting that impaired cardiac function is a risk factor for infertility. Whether this has implications for most women with CHD is unknown. Recent studies report no increase in menstrual cycles required to obtain pregnancy in women with CHD overall [26] and no increased risk of being diagnosed with infertility for women with simple or moderate CHD [21]. Supported by our finding of a similar number of childbirths in women with CHD having at least one childbirth, this might indicate that most women with CHD who choose to have children do not have reduced fertility compared to the reference population.

We expected that improvements in the treatment of CHD and, thereby, a healthier adult CHD population, regional CHD follow-up and available preconceptional evaluations at specialist centers, and the increasing evidence that many women with CHD can go through pregnancy with low risks could lead to more childbirths among women with moderate/severe CHD during the study period. Several explanations for consistently lower birth rates in women with moderate/severe CHD should be considered. According to the risk score for heart disease in pregnancy from the World Health Organization (mWHO), only women with conditions giving the highest cardiac risk (mWHO class V) should avoid pregnancy [11]. However, women with lower risks might have been exposed to less evidence-based advice against pregnancy [27]. Psychiatric disorders represent the most common comorbidity among people with CHD, and together with higher rates of impaired neurocognitive and psychosocial conditions, this can lead to difficulties with establishing relationships and starting a family [28,29]. Concern that pregnancy worsens their health condition, that CHD may have harmful effects on the unborn child, and that their ability to take care of a child would be impaired due to lack of energy or lower life expectancy [[30], [31], [32]] might have led women with CHD to avoid pregnancy. Despite most heart defects having a modest recurrence rate between 2 and 6% [22,33], some women might have avoided pregnancy due to fear of passing the CHD on to their children [[30], [31], [32]].

The findings of this study have several implications. First, women with mild CHD should be encouraged that their prospects of having children do not seem affected by their heart defects. Second, to empower women with moderate/severe CHD to make informed choices on whether to embark on pregnancy, healthcare professionals should address the topic of childbearing in patient education and counseling and provide individualized information on maternal and neonatal risks in pregnancy. Third, healthcare authorities should acknowledge the increasing prevalence of maternal CHD to provide the necessary healthcare to this growing patient group.

## 5 Strengths and limitations

The strength of the present study was the use of individual-level data in a national cohort, including more than 5700 women with CHD, and among those, almost 1300 women with moderate/severe CHD. Linking data from several comprehensive national registries allowed essentially complete ascertainment of CHD, and by the unique personal identification number provided to each Norwegian resident, we can follow the woman from birth through life; therefore, a heart defect could be attributed to her even when not recognized at the time of pregnancy. Norwegian women have access to a universal healthcare system with follow-up during childhood and pregnancy free of charge; therefore, selection bias and loss to follow-up are less likely.

There are some limitations concerning misclassification. Women with mild CHD diagnosed before the study started in 1994 or before registration of out-patient diagnoses in 2008 could have been missed if they had no later medical check-ups or were hospitalized for other reasons, or a silent CHD could have been detected after the study period. As pregnancy might have been the reason for registration or uncovering a silent CHD, we excluded 363 women with diagnoses of CHD from the sole source Medical Birth Registry from analyses of birth rates to avoid bias. The risk of random misclassifications of CHD is minimized by our considerate prioritization of diagnosis according to the women's age at the time of diagnosis and the departments and classification system that originated the diagnosis.

We did not have access to physiological measurements to describe the clinical severity of CHD. However, compared to registrations of a single diagnosis, the hierarchical algorithm allows for combinations of diagnoses to provide specific characteristics and thereby determine the anatomic severity of the CHD diagnosis.

All childbirths in Norway were detected due to the compulsory registration of all births in MBRN, but childbirths abroad were not included. As a smaller proportion of women with CHD than women without heart disease were born abroad (9.8% vs. 15.6%), birth rate differences could be larger if women born abroad also had more childbirths abroad.

Norway's high-quality universal health care system with good outcomes for individuals with CHD may not be generalized to populations with fewer resources. Finally, it was not possible for us to add further observation time beyond 2014 to this study. However, including two decades provides essential information on women with CHD and a trend pointing towards our time.

## 6 Conclusions

In a national cohort of more than 1.6 million women aged 15–45 years and nearly 1.2 million childbirths in Norway over two decades, the prevalence of maternal CHD in childbirth reflected the increasing prevalence of CHD in women in the population.

Compared to women without heart disease, women with moderate/severe CHD were less likely to have children and had a lower mean number of childbirths. For women with mild CHD, the birth rates did not seem affected by the heart defect. Further studies should investigate whether biology, psychosocial conditions, or suboptimal counseling and follow-up from the healthcare system prevent women with moderate/severe CHD from having children.

## Sources of funding

Financial support was provided by the 10.13039/501100008568Norwegian Women's Public Health Association, the Norwegian Association for Children with Congenital Heart Disease, and the 10.13039/100002129Heart Foundation at the 10.13039/501100005036University of Bergen. The funders had no role in the study design, analysis, or manuscript preparation.

## CRediT authorship contribution statement

**Marit Sandberg:** Writing – review & editing, Writing – original draft, Visualization, Project administration, Methodology, Investigation, Funding acquisition, Formal analysis, Data curation, Conceptualization. **Tatiana Fomina:** Writing – review & editing, Validation, Supervision, Formal analysis, Data curation, Conceptualization. **Ferenc Macsali:** Writing – review & editing, Writing – original draft, Supervision, Conceptualization. **Gottfried Greve:** Writing – review & editing, Writing – original draft, Supervision, Conceptualization. **Mette-Elise Estensen:** Writing – review & editing, Writing – original draft, Conceptualization. **Nina Øyen:** Writing – review & editing, Writing – original draft, Visualization, Supervision, Project administration, Funding acquisition, Conceptualization. **Elisabeth Leirgul:** Writing – review & editing, Writing – original draft, Visualization, Supervision, Project administration, Funding acquisition, Conceptualization.

## Declaration of competing interest

The authors declare that they have no known competing financial interests or personal relationships that could have appeared to influence the work reported in this paper.

## Figures and Tables

**figs1:**
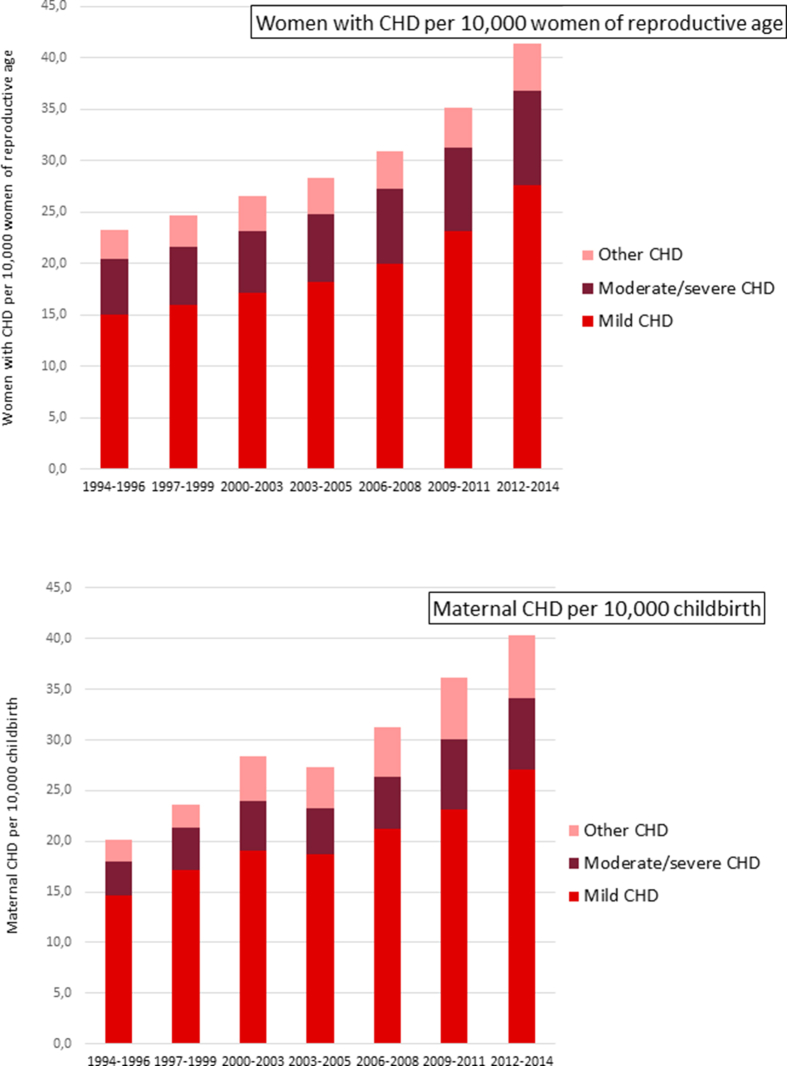
**Supplementary figure 2.** The prevalence of CHD per 10,000 women among 1,644,650 women of reproductive age (15-45 years), and the prevalence of maternal CHD per 10,000 childbirths among 1,183,851 childbirths in Norway 1994- 2014. Abbreviations: CHD, congenital heart disease.
