# Supplementary material for: Time trends and birth rates in women with congenital heart disease; a nationwide cohort study from Norway 1994–2014
Source: Int J Cardiol Congenit Heart Dis. 2024 Mar 30;16:100507. doi: 10.1016/j.ijcchd.2024.100507 (PMC11657671; doi:10.1016/j.ijcchd.2024.100507)

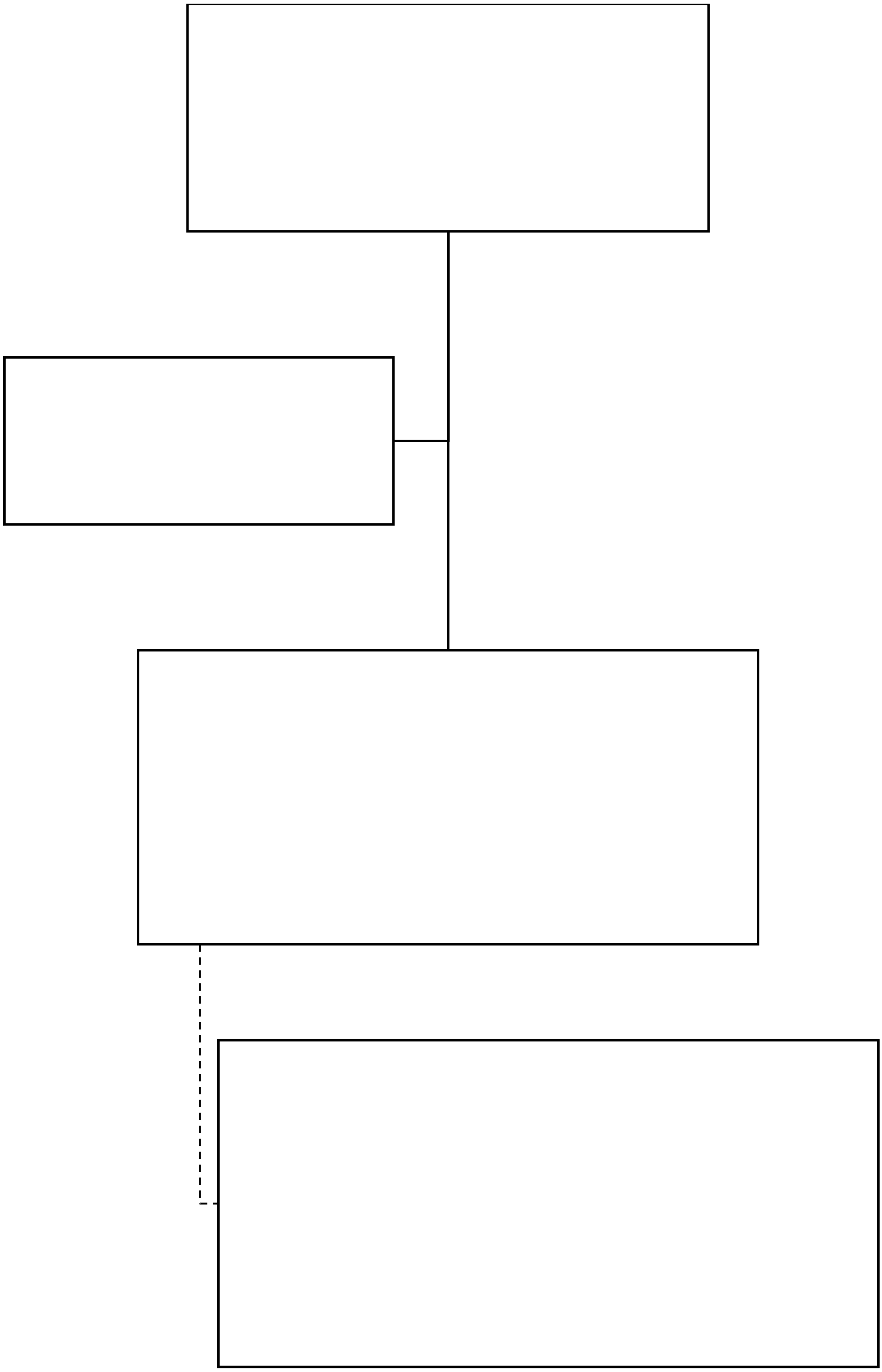

Supplement: Supplementary file 1 [file mmc1.docx]
